# Is the Pharmacokinetics of First-Line Anti-TB Drugs a Cause of High Mortality Rates in TB Patients Admitted to the ICU? A Non-Compartmental Pharmacokinetic Analysis

**DOI:** 10.3390/tropicalmed8060312

**Published:** 2023-06-08

**Authors:** Francisco Beraldi-Magalhaes, Suzanne L. Parker, Cristina Sanches, Leandro Sousa Garcia, Brenda Karoline Souza Carvalho, Amanda Araujo Costa, Mariana Millan Fachi, Marcus Vinicius de Liz, Alexandra Brito de Souza, Izabella Picinin Safe, Roberto Pontarolo, Steven Wallis, Jeffrey Lipman, Jason A. Roberts, Marcelo Cordeiro-Santos

**Affiliations:** 1 Programa de Pós-Graduação em Medicina Tropical, Universidade do Estado do Amazonas, Manaus 69040-000, Brazil; 2 Fundação de Medicina Tropical Doutor Heitor Vieira Dourado, Manaus 69040-000, Brazil; 3 Secretaria de Estado da Saúde do Paraná, Curitiba 80010-130, Brazil; 4 School of Medicine, Faculdades Pequeno Príncipe, Curitiba 80230-020, Brazil; 5 UQ Centre for Clinical Research, The University of Queensland, Brisbane, QLD 4029, Australia; 6 Department of Pharmacy, Campus Centro-Oeste Dona Lindu, Universidade Federal de São João del-Rei, Divinopolis 35501-296, Brazil; 7 Department of Pharmacy, Campus Jardim Botânico, Universidade Federal do Paraná, Curitiba 80210-170, Brazil; 8 Department of Chemistry & Biology, Campus Curitiba, Universidade Tecnológica Federal do Paraná, Curitiba 81280-340, Brazil; 9 Department of Intensive Care Medicine, Royal Brisbane and Women’s Hospital, Brisbane, QLD 4029, Australia; 10 Division of Anaesthesiology Critical Care Emergency and Pain Medicine, Nîmes University Hospital, University of Montpellier, 30900 Nimes, France; 11 Department of Pharmacy, Royal Brisbane and Women’s Hospital, Brisbane, QLD 4029, Australia; 12 School of Medicine, Universidade Nilton Lins, Manaus 69058-040, Brazil

**Keywords:** tuberculosis, antitubercular agents, rifampin, isoniazid, pyrazinamide, ethambutol, pharmacokinetics, biological availability, intensive care, critical care

## Abstract

Background: Patients with tuberculosis (TB) may develop multi-organ failure and require admission to intensive care. In these cases, the mortality rates are as high as 78% and may be caused by suboptimal serum concentrations of first-line TB drugs. This study aims to compare the pharmacokinetics of oral rifampin, isoniazid, pyrazinamide and ethambutol patients in intensive care units (ICU) to outpatients and to evaluate drug serum concentrations as a potential cause of mortality. Methods: A prospective pharmacokinetic (PK) study was performed in Amazonas State, Brazil. The primary PK parameters of outpatients who achieved clinical and microbiological cure were used as a comparative target in a non-compartmental analysis. Results: Thirteen ICU and twenty outpatients were recruited. The clearance and volume of distribution were lower for rifampin, isoniazid, pyrazinamide and ethambutol. ICU thirty-day mortality was 77% versus a cure rate of 89% in outpatients. Conclusions: ICU patients had a lower clearance and volume of distribution for rifampin, isoniazid, pyrazinamide and ethambutol compared to the outpatient group. These may reflect changes to organ function, impeded absorption and distribution to the site of infection in ICU patients and have the potential to impact clinical outcomes.

## 1. Introduction

Tuberculosis (TB) remains one of the world’s deadliest communicable diseases [1]. TB usually affects the lungs but may present acutely in almost any organ system where it can mimic other infectious or non-infectious processes. Up to 16% of TB patients may develop multi-organ failure, including acute respiratory failure and high rates of acute respiratory distress syndrome, sometimes presenting with confluent tuberculous bronchopneumonia. These patients often require admission to intensive care [2,3,4]. TB is a treatable disease, with cure rates around 85% when in the outpatient clinic. However, in intensive care, the mortality rates can reach 78% [5,6,7,8]. The altered pathophysiology experienced by patients in the intensive care unit (ICU) may lead to sub-optimal dosing of first-line antimicrobials used to treat TB and lead to poor patient outcomes [5].

Drug-susceptible TB treatment uses a combination of rifampicin, isoniazid, pyrazinamide and ethambutol in the first two months, followed by four months of rifampicin and isoniazid [9]. The initial phase aims to quickly reduce bacillary population, decreasing contagiousness and patient symptoms [10]. For this early phase to be effective in TB patients admitted to ICU, the pharmacokinetic–pharmacodynamic (PK/PD) targets for all first-line TB antimicrobial must be achieved; otherwise, patient outcomes may be compromised and selective pressure applied for the emergence of antimicrobial resistance [11,12].

Parenteral administration of first-line TB antimicrobials is recommended in ICU [5,13]. However, as this combination is not available intravenously in many of the high TB burden countries, including Brazil, fixed-dose combination tablets are crushed and delivered through a nasogastric tube. Despite the success of using fixed-dose combination tablets in outpatients [14], this strategy may compromise drug absorption and be a cause of high mortality in ICU [15,16]. A comparative analysis of the pharmacokinetics of rifampin, isoniazid, pyrazinamide and ethambutol between ICU patients and outpatients may enable a greater understanding of this relationship.

Therefore, the aim of this study was to compare the primary pharmacokinetic parameters of rifampin, isoniazid, pyrazinamide and ethambutol in patients admitted to the ICU with outpatients, evaluating these results in the context of being a potential cause of mortality.

## 2. Materials and Methods

This paper was completed in accordance with the ClinPK checklist report [17].

### 2.1. Ethics

This study was approved by the Ethics Committee at Fundação de Medicina Tropical Dr. Heitor Vieira Dourado (CEP/FMT-HVD CAAE: 60219916.5.0000.0005). Signed informed consent was obtained from each participant or legal representative for the use of biological materials and publication of data.

### 2.2. Patients and Study Design

This was a prospective open label pharmacokinetic study performed in Amazonas, Brazil, from November 2016 to May 2018. We enrolled individuals ≥ 18 years of age diagnosed with active pulmonary and extrapulmonary TB and who were prescribed fixed-dose combination tablets containing rifampin, isoniazid, pyrazinamide and ethambutol. Patients were considered to have active TB if at least two of the following criteria were met: (1) smear positive for acid-fast bacilli (AFB) or GeneXpert MTB/RIF© (Cepheid, Sunnyvale, CA, USA) on sputum, tracheal aspirate or any other clinical specimen; (2) culture-positive for *Mycobacterium tuberculosis* on sputum, tracheal aspirate or any other clinical specimen; (3) strong clinical suspicion of active TB; or (4) strong radiological evidence of active TB. A strong clinical suspicion of active TB required at least two out of four constitutional symptoms (weight loss with accompanying fever, night sweats, productive cough, loss of appetite for 2 weeks), as well as known TB contact or history of previous pulmonary TB [6].

Patients were recruited at the outpatient clinic or at the ICU of Fundação de Medicina Tropical Dr. Heitor Vieira Dourado in Manaus, Amazonas, Brazil. The diagnosis and treatment were prescribed by the patients’ assistant physician, and if this met the inclusion criteria of the study, they were invited to participate. Patients received fixed-dose combination tablets containing rifampin 150 mg, isoniazid 75 mg, pyrazinamide 400 mg and ethambutol 275 mg following a weight-based dose scheme as follows: for patients weighing 20–35 kg: 2 tablets; 36–50 kg: 3 tablets; and >50 kg: 4 tablets. This weight-based dosing scheme was performed in accordance with the Brazilian Ministry of Health Guidelines as defined at the time of the study. Pregnant women, patients requiring hemodialysis, continuous renal replacement therapy, peritoneal dialysis or those patients whose clinician considered them unsuitable for enrolment were excluded. Clinical and demographic data were recorded for the parameters of body mass index (BMI), weight, renal and liver function, 8 h measured creatinine clearance, blood cell count, sequential organ failure assessment (SOFA) and acute physiology and chronic health evaluation-II (APACHE II) score, human immunodeficiency virus (HIV) status, hepatitis B and C, syphilis, diabetes, comorbidities, concomitant medication in use and antimicrobials used in previous 30 days, occupation, age and sex. The outpatients did not have their SOFA assessed, since they did not show any organ dysfunction. Each ICU patient was assessed daily for their individual requirements for vasopressors and APACHE II and SOFA scores. The outpatients were invited for admission at the Clinical Research Ward for 72 h for directly observed treatment with fixed-dose combination tablets and sample collection. All patients remained in contact with the study staff until the end of the study treatment.

All patients in the ICU group were mechanically ventilated and received fixed-dose combination tablets through a nasogastric tube. Prior to administration, the research nurse crushed fixed-dose combination tablets and suspended them in 20 mL of distilled water and administered the suspension through the nasogastric tube. Afterward, more 20 mL of distilled water was flushed through the nasogastric tube to ensure the ethambutol-containing suspension reached the gastrointestinal tract. Blood samples were collected from each patient immediately prior to parenteral administration of the fixed-dose combination TB treatment and then at 0.5, 1, 2, 4, 6, 8, 12 and 24 h (prior to subsequent dose) on the first and third days of enrollment. Blood samples were collected and immediately stored at 4 °C until centrifugation at 434× *g* for 10 min. Plasma (2 mL) was transferred into a labeled cryotube and stored at −80 °C until analysis.

### 2.3. Drug Assay

Total rifampin, isoniazid, pyrazinamide and ethambutol plasma concentrations were measured simultaneously using a previously validated method [18] with a high-pressure liquid chromatography with a Waters Xevo G2-S QtoF mass spectrometer (Waters Corp., Milford, MA, USA). The validated concentration ranges for each drug were 0.25 to 2 mg/L for rifampicin, 0.2 to 7.5 mg/L for isoniazid, 1 to 40 mg/L for pyrazinamide and 0.2 to 5 mg/L for ethambutol. Bioanalytical method validation guidelines recommend the preparation of a dilution quality control in case of concentrations over the upper limit of quantification. Both quality control and clinical samples are subjected to the dilution process. According to the Brazilian Health Surveillance Agency and the United States Food and Drug Administration, a dilution is considered acceptable if the accuracy and precision of the quality control sample is within 15% of the nominal concentration and <15% of relative standard deviation. This method allows us to measure concentrations above the upper limit of quantification for each drug. The accuracy was calculated as the relative error. Precision was calculated as the relative standard deviation. The intraday accuracy ranged from 0.26 to 13.7%.

### 2.4. Non-Compartmental Pharmacokinetics Analysis

PK parameters were calculated using Pmetrics v.1.5.0 (Laboratory of Applied Pharmacokinetics and Bioinformatics, Los Angeles, CA, USA) in Rstudio (version 0.99.9.3) as a wrapper for R (version 3.3.1), Xcode (version 2.6.2) and the Intel Parallel Studio Fortran Compiler XE 2017. Patients with (1) drug plasma concentrations below the lower limit of quantification, (2) those displaying concentrations that did not decrease over time, or (3) patients with fewer than five serum concentrations for each drug (minimum parameter required by Pmetrics) were excluded from the PK analysis.

### 2.5. Statistical Analysis

A descriptive analysis was performed of the data by means of distribution of frequency and measurements of central tendency. The categorical variables were expressed as frequency and percentage and analyzed using Pearson’s *X*^2^ test or Fisher’s exact test. For numerical variables, a Mann–Whitney test was used. To compare differences between the dosing occasions of the absorption rate constant for the first and second dose in the same group, a Wilcoxon rank test was used. All analyses were performed considering a significance level of 5%, conducted using R software (version 0.99.9.3).

## 3. Results

Thirteen mechanically ventilated patients in the ICU and twenty outpatients were included in this study. Positive culture, sputum smear microscopy for AFB or GeneXpert MTB/RIF© confirmed TB diagnosis in 62% (8/13) of ICU patients and in 85% (17/20) of outpatients. All patients with positive culture had a drug susceptibility test performed. The median time of treatment before blood sampling for the study was 7 days for both ICU patients (IQR = 3–11 days) and outpatients (IQR = 4–12.3 days). The median time between ICU admission and patient sampling was 3 days (IQR = 2–6 days).

The clinical and demographic characteristics were similar in both groups (see Table 1), except for creatinine clearance, which was significantly lower (*p* = 0.02) in ICU patients at 45.8 (range, 0.0–97.5) mL/min compared to outpatients at 114 (range, 86.5–158) mL/min, and median APACHE-II scores were significantly higher (*p* < 0.01) in the ICU group at 28 (range, 20–33) compared to the outpatient group at 5 (range, 3.8–7).

Among the patients coinfected with HIV and requiring intensive care, 8% (n = 1/12) had a CD4 count > 100 cells/mm^3^, 42% (n = 5/12) were receiving antiretroviral therapy (ARVT), and 8% (n = 1/12) had an undetectable viral load. Among outpatients, 75% (n = 15/20) were living with HIV with a median CD4 count of 121 cells/mm^3^ (IQR = 26–184 cells/mm^3^), 25% (n = 5/20) were already receiving ARVT, and 10% (n = 2/20) had an undetectable viral load.

The results of the non-compartmental PK analysis are presented in Table 2. For rifampin, a total of 18 patients were included in the non-compartmental PK analysis, n = 5 patients in ICU and n = 13 outpatients. The area under the curve (AUC) was almost double in ICU patients compared to outpatients, but no difference was found between patient groups for the maximum concentration (C_max_), time to the maximum concentration (T_max_), absorption constant (Ka), clearance, volume of distribution or half-life (t_1/2_). For isoniazid, a total of 29 patients were included in the non-compartmental PK analysis, n = 11 patients in ICU and n = 18 outpatients. No significant difference was seen for any of the PK parameters. For pyrazinamide, a total of 33 patients were included in the non-compartmental PK analysis, n = 13 patients in ICU and n = 20 outpatients. The T_max_, clearance and volume of distribution were significantly lower in ICU patients compared to outpatients (*p* < 0.01 for all three PK parameters). Finally, for ethambutol, a total of 30 patients were included in the non-compartmental PK analysis, n = 10 patients in ICU and n = 20 outpatients. The AUC and t_1/2_ were significantly higher (*p* = 0.01 and *p* < 0.01, respectively), and Ka and clearance were significantly lower (*p* < 0.01 for both PK parameters) in ICU patients compared to outpatients.

Neither outpatients nor ICU patients achieved predetermined PK targets for pyrazinamide. For isoniazid, no outpatient achieved a C_max_ between 3 and 8 mg/L as expected, and only one ICU patient achieved this mark. Ethambutol expected AUC > 23.6 mg·h/L was reached by 5/10 ICU patients and no outpatient (*p* = 0.0032). On the other hand, ethambutol C_max_ at intervals of 2 and 6 mg/L PK target was reached by 6/10 ICU patients and 2/20 outpatients (*p* = 0.013). Finally, for rifampin, 5/5 ICU patients and 7/13 outpatients marked AUC > 24.1 mg·h/L. The C_max_ > 8 mg/L was achieved by 2/5 ICU patients and 5/13 outpatients.

A 30-day mortality of 77% (n = 10/13) was recorded in the ICU patients. Of the three ICU patients who survived at 30 days, one was transferred to the ward and died after 41 days, and the other two patients remained in the ICU and died after 63 and 122 days of TB treatment. None of the outpatient group died, with 89% (n = 16/18) achieving clinical cure. Two patients did not recover from their infection, one due to abandonment of treatment, and the other patient was diagnosed with MDR-TB 35 days after commencement of treatment. The remaining two patients who were recruited in this study were excluded from this analysis, as they relocated to other regions and were lost to follow-up.

## 4. Discussion

This study demonstrated that, based on a non-compartmental analysis, both the clearance and volume of distribution are lower in ICU patients compared to outpatients receiving a fixed-dose combination of rifampin, isoniazid, pyrazinamide and ethambutol. These differences in pharmacokinetics could influence clinical outcomes if they lead to subtherapeutic concentrations.

The results of this study, which is the first article for this drug combination comparing the pharmacokinetics of ICU patients with outpatients (where both patient groups were being treated for *Mycobacterium tuberculosis*)*,* are based on a weight-based dosing regimen administered to ICU patients via nasogastric tube and to outpatients orally. The current PK/PD targets for these first-line TB drugs are uncertain and need further validation; however, the WHO standard fixed-dose combination regimen shows 85% effectiveness in the general population [1]. In our study, all outpatients who completed an oral fixed-dose combination treatment were cured of TB. Based on this, we assumed that outpatients had achieved PK/PD targets for efficacy of rifampin, isoniazid, pyrazinamide and ethambutol and that the PK parameters derived from our outpatient group can be used as a reasonable comparator for ICU patients and to understand the extent of altered pharmacokinetics.

In the ICU patients, a lower clearance was statistically significant for pyrazinamide and ethambutol and with a tendency toward significance (*p* = 0.07) for rifampin. The clearance for isoniazid was highly variable in the outpatient group but numerically higher than the ICU patient group. All these drugs are renally and/or hepatically cleared, and the lower clearance is supported by the ICU patients having a creatinine clearance, which was 50% lower than the outpatients (*p* < 0.02). Most TB patients admitted to the ICU presented with renal and hepatic failure [5], and it is likely that the hepatic and renal dysfunction presented in the ICU patient group in this study was responsible for the lower clearance observed.

The lower volume of distribution In ICU patients presented statistical significance for pyrazinamide only, a tendency toward significance for ethambutol (*p* = 0.06) and numerically lower results for rifampicin and isoniazid. A lower estimated volume of distribution for hydrophilic drugs is unusual in ICU patients [19] and may be due to altered protein binding for acute phase reactant proteins associated with a higher severity of illness in the ICU patients in the study, with all patients exhibiting high APACHE II scores (above 20).

The targets associated with clinical cure for rifampicin are an AUC_(0–24h)_ > 24.1 mg·h/L [20,21] and C_max_ that exceeds 8 mg/L [22]. A C_max_ ranging from 5.8 to 9.0 mg/L, depending on treatment duration, predicts microbiological cure in 97% of patients [11,23]. The AUC in the outpatient group was lower than expected, but all patients in both groups achieved the AUC concentration threshold value of 13 mg·h/L, which is associated with >91% clinical cure [11]. A C_max_ > 6.6 mg/L was achieved in the ICU patient group, but the range was wider and below the target C_max_ (4.1–8.4 mg/L) for some patients in the outpatient group. For isoniazid, a C_max_ > 3 mg/L is considered therapeutic, and a C_max_ higher than 8.8 mg/L is associated with culture conversion after 2 months [11,24,25]. Additionally, AUC/MIC between 31.33 and 6.4 mg·h/L is associated with better outcomes after 12 months of treatment. The literature suggests that isoniazid MICs should be 0.125 mg/L, and, for our patients, a MIC lower than 0.5 mg/L would be enough to reach this target [26]. None of the patients in this study achieved the C_max_ or AUC targets. Effective dosing of pyrazinamide targets a C_max_ > 35 mg/L and an AUC > 363 mg·h/L to achieve microbiological cure [16,27]. There was no difference in C_max_ or AUC in a comparison between the ICU patients and outpatient groups in this study; however, both of the PK parameters were between 6 and 8 times lower than the targets in both groups [22]. Effective dosing of ethambutol targets an AUC_(0–24h)_ of 23.6 and a C_max_ of 2 to 6 mg/L [22,28]. In this study, the AUC of the ICU patients was three times higher than the outpatient group. Not all of the ICU patients achieved these targets, but none of the outpatients achieved either of these targets. In summary, the targets for AUC and C_max_ were achieved in the ICU group for rifampicin (but not all of the outpatient group). None of the ICU or outpatients achieved therapeutic or microbiological targets for isoniazid or pyrazinamide. Only some of the ICU patients achieved targets for ethambutol only (and none of the outpatients). Therefore, since the targets for AUC and C_max_ in the ICU group were either equal to or greater than those in the outpatient group, it is not possible to attribute the 77% mortality in the ICU group to these parameters.

Sepsis is defined as life-threatening organ dysfunction caused by a dysregulated host response to infection [3]. The severity of organ dysfunction is commonly quantified using the sequential organ failure assessment (SOFA) score [3,29]. Considering that patients in the ICU group had an average SOFA score of 10 points, it is clear that their *Mycobacterium tuberculosis*-related sepsis was severe and that higher mortality was likely in these patients.

Although prediction scores, such as APACHE II and SAPS III, are useful in ICU, they often underestimate the mortality and severity of patients with tuberculosis in this setting [30,31]. Age, dehydration, respiratory failure, lowered level of consciousness and blood pressure are directly related to the risk of death in patients with pulmonary TB [32]. Additionally, mechanical ventilation, septic shock, the need for vasopressors, CD4 count and low serum albumin are factors associated with in-hospital mortality of TB patients [5,8,31,33,34]. Nevertheless, not only the acute condition of the disease but also the lack of early diagnosis and consequent delay of treatment initiation, as well as poor adherence to treatment, comorbidities such as smoking, alcohol abuse and diabetes mellitus, or social vulnerabilities, may be related to the worsening of the disease and contribute to ICU admission [4,35,36]. All these issues reinforce the complexity of a TB patient in critical care.

Despite recommendations to perform drug susceptibility testing for rifampin, isoniazid, pyrazinamide and ethambutol, according to the World Health Organization, the results of these tests may have limitations [37]. Moreover, the MIC cut-off for each drug in the fixed-dose combination is not well defined in the literature, and the detection of resistance conferring mutations at *rpoB*, *inhA*, *katG* and *pncA* genes may be more interesting for clinical practice [26,37,38].

Our research had limitations we would like to declare. This study was performed in a single site, which is a referral center for TB/HIV coinfected patients, and our sample is predominantly composed of men living with HIV. Moreover, in individuals without culture confirmation, nontuberculous mycobacteria infection could not be excluded. MIC testing was not available for this study. Neither the ICU patient group nor the outpatient group achieved all a priori C_max_ and AUC targets for isoniazid, pyrazinamide and ethambutol. However, TB treatment does not rely on each drug individually but on its joint action and drug–drug interactions, which were not assessed in our research [39]. Additionally, it is worth noting the high number of exclusions from pharmacokinetics analysis, mainly due to no observed decay in drug plasma concentrations. Finally, concerning the use of a fixed-dose combination therapy, it seems that adsorption to gastrointestinal tube, drug absorption problems and/or auto-induction metabolism may be in place. This was more pronounced for rifampin in the ICU patients. This finding is supported by Perumal et al. [40] who found low rifampicin concentrations in all patients receiving standard weight-based fixed-dose combination therapy by nasogastric tube.

## 5. Conclusions

ICU patients had a lower clearance and volume of distribution for rifampin, isoniazid, pyrazinamide and ethambutol compared to the outpatient group. These may reflect changes to organ function, impeded absorption and distribution to the site of infection in ICU patients and may have the potential to impact clinical outcomes. The targets of AUC and C_max_ were achieved in the ICU group for rifampicin (but not all of the outpatient group). None of the ICU or outpatients achieved therapeutic or microbiological targets for isoniazid or pyrazinamide. Only some of the ICU patients achieved targets for ethambutol only (and none of the outpatients). The parameters of AUC and C_max_ may not be strongly associated with clinical cure. Future research examining the pharmacokinetics of the combination of these anti-TB drugs in the ICU patient population may provide greater insights into optimal dosing.

## Figures and Tables

**Table 1:** Clinical and demographic data.

Characteristics	ICU (n = 13)	Outpatients (n = 20)	*p*-Value
Age (yrs)	32 (30–52)	40 (33–46)	0.53
Gender (Male/Female)	10 (77%)/3 (33%)	16 (80%)/4 (20%)	1.00
Weight (kg)	52.5 (46.1–60.0)	58.4 (53.1–67.0)	0.14
SOFA score	10 (6.3–12.0)	-	
**APACHE II score**	**28 (20–33)**	**5 (4–7)**	**<0.01**
HIV, n (%)	12 (92%)	15 (75%)	0.42
**Creatinine clearance (mL/min)**	**45.8 (0.0–97.5)**	**114 (86.5–158)**	**0.02**

**Table 2:** Rifampin, isoniazid, pyrazinamide and ethambutol pharmacokinetic parameters estimated using a non-compartmental analysis.

**Rifampin**	**Parameter**	**ICU (n = 5)**	**Outpatients (n = 13)**	***p*** **-Value**
	AUC (mg·h/L)	46.6 (40.6–77.4)	25.1 (21.2–31.5)	**<0.01**
	C_max_ (mg/L)	7.3 (6.6–10.1)	6.7 (4.1–8.4)	0.33
	Ka (h^−1^)	0.12 (0.09–0.25)	0.35 (0.25–0.49)	0.20
	CL (L/h)	9.35 (6.78–13.7)	20.7 (16.4–24.7)	0.07
	T_max_ (h)	2 (2–4)	2 (2–4)	0.80
	Vd (L)	59.2 (53.1–78.2)	83.6 (77.5–102.1)	0.33
	t_1/2_ (h)	6.0 (4.1–7.5)	2.0 (1.4–2.7)	0.19
**Isoniazid**	**Parameter**	**ICU (n = 11)**	**Outpatients (n = 18)**	***p*** **-Value**
	AUC (mg·h/L)	15.2 (6.8–27.8)	14.4 (5.43–31.0)	0.95
	C_max_ (mg/L)	0.70 (0.29–1.6)	0.80 (0.51–1.10)	0.72
	Ka (h^−1^)	0.01 (0.01–0.06)	0.35 (0.25–0.49)	0.20
	CL (L/h)	8.60 (2.51–14.5)	33.9 (3.36–47.8)	0.22
	T_max_ (h)	1 (0.5–3)	2 (1–2)	0.76
	Vd (L)	1350 (416–2170)	1740 (842–1750)	1.00
	t_1/2_ (h)	125 (12–560)	24 (22–52)	0.84
**Pyrazinamide**	**Parameter**	**ICU (n = 13)**	**Outpatients (n = 20)**	***p*** **-Value**
	AUC (mg·h/L)	46.2 (12.4–143)	58.4 (35.0–74.6)	0.65
	C_max_ (mg/L)	3.5 (2.8–14)	7.3 (5.6–8.5)	0.37
	Ka (h^−1^)	0.1 (0.08–0.1)	0.1 (0.09–0.2)	0.25
	CL (L/h)	8.23 (5.47–9.82)	21.2 (16.5–31.0)	**<0.01**
	T_max_ (h)	1 (1–2)	2 (2–4)	**<0.01**
	Vd (L)	94.7 (81.0–113)	221 (163–264)	**<0.01**
	t_1/2_ (h)	7.0 (7.0–8.4)	5.4 (4.6–7.3)	0.26
**Ethambutol**	**Parameter**	**ICU (n = 10)**	**Outpatients (n = 20)**	***p*** **-Value**
	AUC (mg·h/L)	22.0 (6.82–36.1)	6.46 (4.84–8.73)	**0.01**
	C_max_ (mg/L)	2.3 (1.0–3.1)	1.2 (1.1–1.6)	0.13
	Ka (h^−1^)	0.04 (0.03–0.08)	0.22 (0.15–0.28)	**<0.01**
	CL (L/h)	25.1 (10.4–61.8)	131 (117–159)	**<0.01**
	T_max_ (h)	2 (2–5.5)	3 (2–4)	0.96
	Vd (L)	486 (399–811)	811 (584–1050)	0.06
	t_1/2_ (h)	16 (9–24)	3.2 (2.5–4.6)	**<0.01**

Data expressed as median and interquartile range (IQR) and Mann–Whitney test.

## Data Availability

The data presented in this study are available on request from the corresponding author.
